# Artificial intelligence and machine learning for organizational and system-level functions in primary health care: A scoping review protocol

**DOI:** 10.1371/journal.pone.0329426

**Published:** 2026-09-25

**Authors:** Pablo Galvez-Hernandez, Li-Anne Audet, Zahra Shakeri, Walter P. Wodchis

**Affiliations:** 1 Department of Health and Society, University of Toronto Scarborough, Toronto, Ontario, Canada; 2 Institute of Health Policy Management and Evaluation, Dalla Lana School of Public Health, University of Toronto, Toronto, Ontario, Canada; 3 Institute for Better Health, Trillium Health Partners, Toronto, Ontario, Canada; Khoy University of Medical Sciences, IRAN, ISLAMIC REPUBLIC OF

## Abstract

**Objective:**

Primary health care (PHC) is the cornerstone of health systems worldwide, yet faces growing pressures from aging populations, workforce shortages, and constrained resources. While artificial intelligence (AI) and machine learning (ML) are increasingly used for clinical tasks such as diagnostics and decision support, their potential to address organizational and system-level processes remains underexplored. This scoping review maps AI and ML applications targeting non-clinical, organizational, and system-level functions in PHC, examining techniques employed, application purposes, data sources, and implementation maturity, and identifying evidence gaps to inform future research.

**Methods:**

This scoping review will be conducted using the Arksey and O’Malley framework and reported according to PRISMA-ScR. We will search Ovid MEDLINE, EBSCO CINAHL, Ovid Embase, Cochrane Library (Wiley), Ovid PsycINFO, and IEEE Xplore, as well as grey literature sources, from January 2010 to December 2025, with a pre-submission update. Two independent reviewers will screen titles, abstracts, and full texts. Eligible studies include empirical research describing, evaluating, implementing, or developing AI and ML applications at the meso-level (organizational, e.g., care scheduling, population stratification) and macro-level (system-wide, e.g., workforce planning, funding allocation) of PHC. Studies on micro-level clinical applications, non-empirical research, and secondary literature will be excluded. Applications will be classified using a two-layer taxonomy (AI technique family and core algorithm) and mapped to PHC building blocks adapted from WHO operational frameworks. Synthesis will examine technique-building block intersections, application purposes, data sources, implementation maturity, and evidence gaps. The protocol is registered in Open Science Framework (osf.io/wzj5x).

**Conclusions:**

This review will provide a timely synthesis of AI and ML applications at organizational and system levels of PHC, identifying which building blocks have been addressed, which remain underexplored, and where the field stands in terms of implementation readiness. The findings will inform a targeted research agenda and provide actionable evidence for decision-makers seeking to leverage AI and ML for PHC strengthening.

## Background

Applications of Artificial Intelligence (AI) and Machine Learning (ML) in healthcare have grown exponentially over the past decade [1]. AI can be defined as a broad field focused on systems that exhibit intelligent behavior by deriving knowledge from data [2]. Machine learning (ML), commonly considered a subfield of AI, uses algorithms to learn associations with predictive power from data examples through statistical models [2]. These tools enable the analysis of large, complex data to uncover patterns and predict outcomes, offering promising advancements in how healthcare is delivered.

Most studies on AI and ML in healthcare have focused on their clinical applications, including diagnostic tools, chatbots, and predictive analytics embedded in healthcare IT infrastructure for patient care delivery [3,4]. Additionally, there is increasing interest in population-based applications in public health, often referred to as precision population health management, which uses advanced analytics (e.g., AI, machine learning, and predictive modeling) to stratify populations and tailor interventions based on clinical, behavioral, and social data, aiming to deliver targeted, equitable care at scale [5]. Numerous empirical data have shown that AI and ML can assist clinicians to make better decisions, early diagnosis and patient monitoring [6,7]. These benefits have also been synthesized in systematic reviews, which highlighted the advantages of AI and ML for patients, families, and healthcare professionals in various healthcare contexts [8,9].

From a systems thinking perspective, effective health care delivery is not only a function of clinical decision-making but is fundamentally shaped by the structural and organizational processes that support it, including governance, financing, workforce deployment, and service planning [10]. These meso-level (organizational and team-level) and macro-level (regional, provincial, and national regulatory and funding structures) dimensions constitute the infrastructure upon which clinical care operates [11]. While AI and ML have demonstrated transformative potential for clinical tasks, the capacity of these technologies to address the organizational and system-level challenges that constrain health care delivery has received comparatively limited attention in the literature.

Within health systems, Primary Health Care (PHC), a cornerstone of efficient health systems and universal health coverage, faces persistent challenges in sustaining high-quality service delivery [12]. Examples of structural and process-related challenges include resource allocation favouring specialized care; difficulties in planning and funding PHC strategies that involve multisectoral collaboration; adapting care models to local contexts; inadequate workforce planning amid shortages; inefficiencies in payment and purchasing systems; and deploying integrated monitoring and evaluation frameworks [12,13]. These challenges are compounded by an aging population with complex multimorbidity and growing social needs, placing additional strain on PHC systems [14]. While economic and workforce resource constraints present a major burden, existing infrastructures could be strengthened through more effective resource allocation, streamlined care pathways, needs-based service and workforce planning, and better integration supported by timely data [15,16]. AI and ML might have the potential to support these improvements; however, their role in addressing these challenges remains unclear and has not been comprehensively synthesized in existing reviews.

Some empirical research has explored the application of AI and ML beyond clinical encounters in primary care, including population-level risk stratification [17], patient phenotyping and panel size optimization to improve provider workload distribution [18], and appointment scheduling [19]. However, a comprehensive understanding of AI and ML applications across the broader organizational and system-level dimensions of primary care remains absent, as existing reviews have focused predominantly on micro-level applications involving individual patients, providers, and their clinical interactions. For example, Abbasgholizadeh et al. mapped AI applications in community-based PHC and found that diagnosis, risk stratification, disease surveillance, and clinical decision support were the dominant use cases, relying primarily on machine learning and natural language processing [8]. These existing syntheses confirm the dominance of micro-level clinical applications in the PHC AI/ML literature and underscore the need of reviews addressing the organizational and system-level domains targeted by this protocol. While AI techniques are increasingly being applied to structural challenges such as strategic purchasing and funding allocation in health systems [20], evidence of such applications within the PHC context specifically remains limited.

Addressing this gap is essential for understanding how AI and ML may support core PHC system functions, including governance, resource allocation, workforce planning, and intersectoral coordination. This scoping review aims to: (i) map and characterize current AI and ML applications in organizational and system-level dimensions of PHC; (ii) identify the type of data sources and methods used to develop and apply AI/ML models; (iii) examine reported outcomes, strengths, and limitations; and (iv) identify evidence gaps and future directions.

## Methods

This scoping review follows the five stages proposed by Arksey and O’Malley: (i) identifying the research questions; (ii) identifying relevant studies; (iii) study selection; (iv) charting the data; and (v) collating, summarizing and reporting the results [21]. A scoping review was selected because it is designed to map the breadth of a heterogeneous evidence base, identify key concepts, and clarify the boundaries of an emerging research area [21]. Results will be reported according to the PRISMA Extension for Scoping Reviews (PRISMA-ScR) checklist [22]. The analytical approach integrates a classification taxonomy for AI/ML techniques that captures the type of methods employed and a set of PHC building blocks adapted from WHO operational and measurement frameworks [12,15], which will enable a systematic cross-mapping of techniques to PHC building blocks, which constitutes the primary analytical output of this review. This protocol has been registered in Open Science Framework (OSF) (osf.io/wzj5x). In addition, a preprint version was posted on medRxiv through the journal’s facilitated preprint posting service during the manuscript submission process. Ethics approval was not required for this study because human subjects are not involved.

### Scoping review stages

#### Stage 1: Defining the research questions.

The review will be guided by the following research questions: (i) What are the current applications of AI and ML at the organizational and system-levels of PHC as reported in the literature? (ii) What types of data sources (e.g., electronic health records, administrative databases, national registries, survey data) have been used to develop and apply AI and ML models in this context? (iii) What are the strengths and limitations of these applications as reported by study authors? (iv) What gaps exist in the current evidence base in terms of underused AI and ML techniques across PHC building blocks, and implementation maturity?

#### Stage 2: Identifying relevant studies.

Studies will be searched in Ovid MEDLINE, EBSCO CINAHL, Ovid Embase, Cochrane Library (Wiley), Ovid PsycINFO, and IEEE Xplore. A grey literature search will be conducted in OpenGrey, Google Scholar, and ProQuest Dissertations & Theses Global. Reference lists of included articles and relevant literature reviews will also be searched to identify additional studies [21]. The search will be restricted to English-language publications from January 2010 to December 2025, with a final search update conducted prior to manuscript submission, to ensure the review reflects the most current evidence available at the time of publication. The start date was chosen to capture the period of rapid expansion of AI and ML in healthcare, marked by the widespread adoption of deep learning techniques in the early 2010s [23].

A search strategy was iteratively developed following guidance from the Joanna Briggs Institute [24] and the Cochrane Handbook for Systematic Reviews of Interventions [25]. The strategy was informed by preliminary searches, comparison with search strategies from relevant published systematic and scoping reviews on AI/ML in health care, and term analysis of five relevant articles identified through an initial limited search in MEDLINE (Ovid) using the terms “artificial intelligence,” “machine learning,” and “primary health care.” Titles, abstracts, keywords, and index terms from these articles were examined using the Yale MeSH Term Analyzer [26] to identify additional controlled vocabulary and free-text terms. The resulting strategy combined keywords and index terms for two core concepts, AI/ML and primary health care, using Boolean operators (Table 1). It was then reviewed for sensitivity during consultation with an expert librarian at the University of Toronto and adapted to the syntax and controlled vocabulary of each database. The five hand-searched articles were used as a known-item check to confirm retrieval of relevant studies. The term “data mining” was considered but not included as a separate term because of substantial overlap with machine learning.

**Table 1:** Search strategy for Ovid MEDLINE.

#	Search terms
1	Artificial Intelligence/
2	Machine Learning/
3	Sentiment Analysis/
4	Deep Learning/
5	Natural Language Processing/
6	neural networks, computer/
7	Decision Support Systems, Clinical/
8	(artificial intelligence or machine learning or sentiment analysis or natural language processing or neural networks or deep learning or supervised learning or unsupervised learning or semi-supervised learning or reinforcement learning).tw,kf.
9	(expert system* or fuzzy logic or knowledge-based system* or knowledge based system* or Bayesian network* or large language model* or generative artificial intelligence or generative AI or transformer model*).tw,kf.
10	(predictive model* or prediction model* or automated decision* or computer-aided or computer aided or computer-assisted or computer assisted).tw,kf.
11	1 or 2 or 3 or 4 or 5 or 6 or 7 or 8 or 9 or 10
12	Primary Health Care/
13	Community Health Services/
14	Home Care Services/
15	Family Practice/
16	(primary care or primary healthcare or primary health care or primary practice* or general practice* or family practice* or ambulatory care or community care or community health or home care).tw,kf.
17	((primary or community or family) adj2 (health* or healthcare* or health care* or practice* or medicine)).tw,kf.
18	12 or 13 or 14 or 15 or 16 or 17
19	11 and 18
20	limit 19 to english
21	limit 20 to yr="2010 - 2025"

#### Stage 3: Study selection.

All references will be uploaded to Rayyan software for deduplication and screening. Two independent reviewers (PGH, LA) will conduct title, abstract, and full text screening using a detailed protocol outlining the eligibility criteria. To validate the screening process, both reviewers will first simultaneously screen a sample of 25 articles as a training exercise [21]. A calibration exercise will then be conducted, with both reviewers independently screening 20% of articles at each stage, targeting an inter-rater reliability above 80% [27]. Discrepancies will be resolved through periodic meetings to discuss decision rationales, address inconsistencies, and reach consensus, with the involvement of a third reviewer as tie-breaker when necessary.

The eligibility criteria are defined a priori, drawing from the conceptual definitions in Table 2. Inclusion criteria are: (i) published and unpublished primary studies that (ii) describe, evaluate, implement, or develop AI/ML applications (iii) at the organizational and system-levels of PHC systems; for example, clinic management, workforce planning, primary care policy analysis, or resource allocation across services. Unpublished primary studies include dissertations, theses, conference papers and proceedings, and reports. AI methods in this review primarily refer to computational techniques that enable learning of data-driven decision-making; knowledge-driven AI (e.g., expert systems) is also included where it appears in the context of PHC systems. All categories and techniques of AI and ML are eligible (e.g., supervised, unsupervised, semi-supervised, reinforcement learning, deep learning, natural language processing).

**Table 2:** Conceptual definitions underpinning eligibility criteria and the analytical classification framework.

Concept	Categories and definitions
AI technique families	For the purposes of classification and synthesis, AI techniques are grouped into four families based on their primary analytical approach [28]: *(i) Traditional ML:* Algorithms used to classify and predict structured data; (ii) *Deep learning:* Neural networks with multiple hidden layers to analyze high-dimensional patterns in large, complex datasets; (iii) *Natural language processing (NLP):* Techniques for mining, extracting, or classifying information from unstructured text.; (iv) *Other AI/ Hybrid:* Rule-based systems, expert systems, fuzzy logic, or mixed approaches that do not fit the above categories.
Core algorithms	The specific model(s) used for the main analytical task, such as logistic regression, random forest, support vector machines, decision trees, k-nearest neighbors, gradient boosting, deep neural networks, k-means clustering, or topic modelling [2,29]. Studies may employ multiple core algorithms.
Machine learning approaches	ML algorithms are categorized by learning approach [28,29]: *(i) Supervised learning:* Algorithms that are trained on labeled data to map inputs to outputs for classification or regression; *(ii) Unsupervised learning:* Algorithms that analyze unlabeled data to uncover hidden patterns or structures.;*(iii) Semi-supervised learning:* Algorithms trained on a small amount of labeled data combined with a larger pool of unlabeled data; *(iv) Reinforcement learning:* algorithms that learn through interaction with an environment, using feedback (rewards or penalties) to optimize actions.
Application purpose	The functional objective of the AI/ML application, classified as: prediction, classification, clustering/segmentation, optimization, monitoring/surveillance, decision support, or other.
Primary Health Care and Primary Care	*Primary health care (PHC):* A comprehensive, society-wide strategy aimed at organizing and enhancing national health systems to deliver health and well-being services closer to communities [30]. *Primary care:* The settings that constitute the initial point of contact with the healthcare system, such as primary care centers, community health centers, and community pharmacies [30].
Meso and macro levels of PHC systems	*Meso level*: The organizational or local institutional level of PHC systems, including organizational dynamics, team-level processes, and structural elements such as facility budgets and local intersectoral partnerships [31]. *Macro level:* Broader systemic influences, including national and provincial policies, scope of practice regulations, and system-wide budgeting and resource allocation [31].

Note: Categories are not mutually exclusive (e.g., deep learning is frequently applied within NLP tasks); where overlap occurs, studies will be classified by their primary technique and learning approach. These definitions inform both the inclusion/exclusion criteria for study selection and the classification framework used during data extraction and synthesis.

Exclusion criteria are: (i) non-empirical publications (e.g., commentaries, editorials); (ii) secondary studies such as literature reviews and meta-analyses; (iii) studies reporting micro-level AI and ML applications in PHC (e.g., diagnostic tools, patient-facing digital technologies); (iv) studies conducted in settings other than PHC, such as clinical, public health, or epidemiological research focused solely on the efficacy of technologies for individual patients or on measuring population health profiles.

#### Stage 4: Charting the data.

The data charting process will involve a training phase, a reliability check, and full extraction. A pilot test will be conducted on 10% of included studies to refine the charting form prior to full extraction. One reviewer (PGH) will chart the data, which will be independently verified by a second reviewer (LA). Discrepancies will be resolved through discussion or by a third reviewer if necessary.

The following information will be charted for each included study across four domains: *(i) Study characteristics:* title, author(s), year of publication, country, source, study design, study objectives and funding source. *(ii) AI/ML application:* AI technique family (Traditional ML, deep learning, NLP, or other AI/Hybrid); core algorithm(s) (the specific models used for the main analytical task, e.g., random forest, logistic regression); ML learning approach (supervised, unsupervised, semi-supervised, or reinforcement); application purpose (prediction, classification, clustering/segmentation, optimization, monitoring/surveillance, decision support, or other); and primary data type (structured EHR/claims, unstructured text, administrative data, survey data, images, or mixed). *(iii) Primary system context:* PHC system level (meso, macro, or both); type of setting (e.g., primary care centers, community health centers); target population; and PHC building block, as defined in Table 3. *(iv) Outcomes and appraisal:* implementation stage (development/validation only, pilot tested, or fully deployed); main findings; and strengths and limitations of the AI/ML application as reported by the study authors.

**Table 3:** Organizational and system-level building blocks of Primary Health Care, adapted from the WHO Operational Framework for PHC [12] and the WHO Primary Health Care Measurement Framework [15].

PHC Building block	Description and examples	PHC Level
Workforce planning & distribution	Predicting workforce needs, staff retention and turnover, rural and urban distribution, workload and panel sizing, staff scheduling and allocation.	Macro/Meso
Funding and resource allocation	Health system financing models, cost prediction, budget allocation, payment system design, case-mix classification, efficiency analysis.	Macro/Meso
Service planning and demand estimation	Forecasting service demand, estimating population-level service needs, planning population-based interventions, home and community-based service planning.	Macro/Meso
Population stratification and risk management	Risk stratification for care management, patient segmentation and complexity scoring, panel management, referral optimization and triage, care prioritization.	Meso
Quality monitoring and safety	Quality indicator extraction, patient safety incident classification, performance monitoring, clinical guideline adherence assessment, patient feedback analysis.	Macro/Meso
Digital health infrastructure and data	Clinical data quality improvement, de-identification, medication and diagnostic coding, health information system development, teleconsultation classification.	Meso
Community engagement and equity	Community health worker programs, social needs screening, health equity research, social determinants of health integration, community outreach, intersectoral collaboration planning.	Macro/Meso

#### Stage 5: Collating, summarizing, and reporting the results.

Extracted data will be coded and categorized according to the classification framework defined in Table 2 and mapped to the PHC building blocks defined in Table 3. To organize the mapping of AI/ML applications to non-clinical PHC functions, we defined seven organizational and system-level building blocks (Table 3), adapted from two WHO frameworks: the Operational Framework for Primary Health Care [12] and the Primary Health Care Measurement Framework [15]. These frameworks identify structural and process-level levers for strengthening PHC systems, including workforce deployment, financing, service planning, quality improvement, digital health infrastructure, and community engagement. While the original frameworks encompass a broader set of PHC dimensions, we consolidated those pertaining to organizational and system-level functions into seven building blocks that capture the non-clinical domains to which AI/ML could be applied. Each study will be assigned one primary building block based on the stated purpose of the AI/ML application, guided by the question: “What PHC function does this application directly improve or enable?” Downstream or indirect effects will not be used for assignment.

Using this framework, extracted data will be synthesized in three components: First, a descriptive overview will present temporal trends, geographic distribution, publication venues, PHC settings, and system levels. Second, the landscape analysis will cross-tabulate AI technique families against PHC building blocks, presented as a heat map in which cell size or color intensity reflects the number of studies and empty cells identify research gaps. Application purposes will be mapped to building blocks with illustrative examples from included studies. Third, a methodological quality snapshot will summarize across all included studies the proportion reporting a validation strategy, performance metrics, external validation, and use of publicly available data or code. Findings will be presented narratively, organized by PHC building block, and supported by figures and summary tables. Author-reported strengths and limitations will be compiled, categorized by building block, and analyzed thematically. A dedicated gap analysis section will identify underexplored PHC building block-technique intersections and inform a targeted research agenda. The review process will be documented using the PRISMA-ScR flow diagram and the PRISMA-P checklist (S1 File). Consistent with the aims of a scoping review of mapping the breadth and nature of a research area rather than evaluate intervention effectiveness, a formal risk of bias assessment will not be conducted [21].

## Discussion

This scoping review protocol outlines a structured approach to mapping AI and ML applications at the organizational and system levels of PHC. The review has completed Stage 3 (study selection), and completion of the full scoping review is expected by July 2026. By synthesizing evidence from published and unpublished literature across six databases and grey literature, the review will characterize how AI and ML have been applied to non-clinical PHC functions, including workforce planning, resource allocation, service demand estimation, quality monitoring, and community engagement, and identify where evidence is accumulating and where significant gaps remain. This focus extends beyond the predominantly micro-level clinical applications explored in previous reviews [8,19], addressing a largely uncharted area in health services and policy research.

The review is anticipated to make three contributions. First, the cross-tabulation of AI/ML technique families against PHC building blocks will produce the first structured map of technique-area intersections at the organizational and system levels of PHC, identifying both populated and empty cells that can directly inform a targeted research agenda. Second, by classifying implementation stage and methodological approaches, the review will characterize the field’s readiness for real-world deployment, which has direct relevance for health system decision-makers weighing AI/ML investments. Third, the building blocks taxonomy and classification framework developed for this review may serve as reusable tools for future syntheses in this domain.

Some limitations are anticipated in this scoping review. The restriction to English-language publications may exclude relevant research from regions where PHC literature is published in local languages, particularly in Latin America, East Asia, and Francophone Africa, where primary care systems may be organized differently and where AI/ML applications to system-level challenges could yield distinct insights. Additionally, the use of specific “primary health care” terminology may limit the capture of studies from contexts where equivalent first-level care services use different nomenclature. Additionally, although the search strategy was developed iteratively with librarian consultation and informed by published AI/ML search strategies, the absence of a librarian or information specialist as a co-author may have limited the comprehensiveness and reporting quality of the search methods, as librarian involvement has been associated with higher-quality search reporting in knowledge synthesis studies [32]. Finally, this scoping review will prioritize breadth of mapping over depth of outcome evaluation to identify what exists and where gaps lie, but will not assess the effectiveness of individual AI/ML applications.

Despite these limitations, this review will provide a comprehensive and timely synthesis of AI and ML applications at understudied levels of PHC. The findings will contribute to health services and policy research by identifying evidence gaps, characterizing methodological patterns, and informing recommendations to strengthen the organizational and structural capacity of PHC systems.

## Supporting information

S1 File PRISMA-P checklist. (DOCX)
